# Language and Culture in Health Literacy for People Living with HIV: Perspectives of Health Care Providers and Professional Care Team Members

**DOI:** 10.1155/2016/5015707

**Published:** 2016-06-02

**Authors:** Keitshokile Dintle Mogobe, Sheila Shaibu, Ellah Matshediso, Motshedisi Sabone, Esther Ntsayagae, Patrice K. Nicholas, Carmen J. Portillo, Inge B. Corless, Carol Dawson Rose, Mallory O. Johnson, Allison Webel, Yvette Cuca, Marta Rivero-Méndez, Solymar S. Solís Báez, Kathleen Nokes, Darcel Reyes, Jeanne Kemppainen, Paula Reid, Lucille Sanzero Eller, Teri Lindgren, William L. Holzemer, Dean Wantland

**Affiliations:** ^1^Faculty of Health Sciences, University of Botswana, Gaborone, Botswana; ^2^School of Nursing, University of Botswana, Gaborone, Botswana; ^3^HIV and AIDS Coordination Office, University of Botswana, Gaborone, Botswana; ^4^Global Health and Academic Partnerships, Brigham and Women's Hospital and MGH Institute of Health Professions, Boston, MA 02120, USA; ^5^UCSF School of Nursing, San Francisco, CA 94143-0608, USA; ^6^MGH Institute of Health Professions, Boston, MA 02129, USA; ^7^UCSF, San Francisco, CA 94105, USA; ^8^Bolton School of Nursing, Case Western Reserve University, Cleveland, OH 44122, USA; ^9^University of Puerto Rico, San Juan, PR, USA; ^10^Center for Nursing Research, University of Puerto Rico-Recinto de Ciencias Medicas, San Juan, PR, USA; ^11^Hunter College, CUNY, New York, NY 10010, USA; ^12^HELP/PSI, Yonkers, NY 10701, USA; ^13^University of North Carolina Wilmington, Wilmington, NC 28403, USA; ^14^School of Nursing, University of North Carolina Wilmington, Wilmington, NC 28403-5995, USA; ^15^Rutgers University School of Nursing, Newark, NJ 07102, USA

## Abstract

Low health literacy has been linked to inadequate engagement in care and may serve as a contributor to poor health outcomes among people living with HIV and AIDS. The purpose of this paper was to examine the perspectives of health care providers and professional care team members regarding health literacy in HIV disease. A secondary data analysis was conducted from a qualitative study aimed at understanding factors that help an HIV positive person to manage their HIV disease. Data were collected from sites in Botswana, the US, and Puerto Rico. In the parent study, data were collected through focus group discussions with 135 people living with HIV, 32 HIV health care providers (HCPs), and 39 HIV professional care team members (PCTMs). SPSS was used to analyze quantitative data while ATLAS.ti was used to analyze qualitative data. The findings from analyses of the perspectives of HCPs/PCTMs suggested that linguistic and cultural factors were important themes in the exchange of HIV information between health care providers and PLHIV. These themes included ineffective communication, health seeking behavior, cultural facilitators, and complementary and alternative/traditional healing methods. Thus, this study suggests that language and culture have a major role in health literacy for PLHIV.

## 1. Background

Defined as an individual's ability to access, process, and comprehend health-related information with the goal of making appropriate decisions [1], the concept of health literacy gained momentum in the US in the 1990s [2]. The Institute of Medicine conceptualized health literacy as a “set of individual capacities in the four domains of cultural and conceptual knowledge, speaking and listening skills, writing and reading skills and numeracy” (IOM). Low health literacy has been noted as a risk factor for poor patient outcomes, particularly in patients living with HIV disease [3–6]. Researchers, programmers, and policy makers have realized that, for patients to meaningfully participate in their self-care, health providers must recognize and respect the patient's right to receive oral and written information about his or her health condition and its management in a way that he or she can comprehend [7–9].

Health literacy influences patient outcomes at three critical junctures of access to health care, interaction between patient and health care provider, and self-care [10]. Persons with HIV/AIDS who have low health literacy are more likely to have poor access to health services compared with high health literacy and are at a greater risk for poor health outcomes [11]. For people living with HIV, adequate health literacy is critical for treatment adherence and for promoting healthy behaviors in their daily lives. In order to achieve and maintain HIV viral suppression, adherence to HIV treatment regimens requires a constant, near-perfect medication adherence rate for many medications [12]. Medication adherence is therefore a critical issue because poor rates of adherence can lead to unsuccessful viral suppression, resistance to medication, opportunistic infections, overall poor health, decreased quality of life, and potentially death [13, 14].

Various sociodemographic characteristics may influence health literacy. Women have been found to be less able than men to follow medication instructions and answer questions about their HIV treatment regimen [15]; a high perception of social stigma has been a significant independent predictor of poor medication adherence for those individuals with low health literacy [10]; individuals with low literacy were significantly less likely than those with high health literacy to have taken medications at necessary adherence levels [16]; and individuals with low health literacy were less able to describe CD4 count and viral load and correctly identify medications in their regimen than those with high health literacy [17, 18].

Patient and provider communication varies with levels of health literacy as the person with low health literacy may not know what questions to ask or may not fully comprehend what the provider is communicating. Researchers estimate the proportion of people living with HIV/AIDS who demonstrate lower or marginal health literacy levels to be around 18–20% [3, 4, 16, 19]. There are inconsistent findings on the association between health literacy and medication adherence among PLHIV. Several authors showed that lower functional health literacy is related to poorer adherence [16, 17, 20]. However, others have found that lower functional health literacy was not associated with lower medication adherence [11, 21, 22].

Language and culture play a role in clients' understanding of health information [23]. According to Webster's II dictionary, the term “language” refers to a system of words formed from a combination of voice sounds, gestures, and written symbols used by people of a particular country or by a group of people with a shared history or set of traditions to communicate thoughts and feelings. Language is therefore used to transmit culture from one generation to the other. Inability to read, write, or speak a certain language affects one's health literacy.

It is widely understood that it is critical to use simpler language for those who have low literacy because reading levels influence the ability to understand complex health conditions. Contextual language refers to the use of language associated with someone's life experiences, whereas the use of technical language is referred to as jargon. The speed at which an individual speaks as well as the number of thoughts communicated at a time while people interact is referred to as structural characteristics of dialogues/conversations [24].

The effects of low health literacy might be more pronounced among PLHIV from minority groups than those from the dominant culture because of the interactions between literacy and language as a cross-cultural barrier [25]. For instance, a San Francisco born patient with low health literacy and HIV might be able to communicate with health care providers, navigate the health care system, and self-manage the HIV and other comorbidities more effectively than a recent migrant who also has low literacy and HIV [25]. The US Department for Health and Human Services (USDHHS) (2011), in a study of ten HIV services that treat predominantly Latinos, suggested that language differences between providers and clients serve as a deterrent to accessing such services. According to Singleton and Krause [25], language and culture provide a context for the acquisition and application of health literacy skills.

Healthy People 2020 defines health literacy as “the ability to understand basic health information and services needed to make appropriate health care decisions” [9, 26]. Other definitions suggest that a person must first be able to obtain and interpret health information and services [1]. Furthermore, the US Institute of Medicine (IOM) notes that health literacy is not solely determined by a patient's ability to access and use health information and services; it is also impacted by the way in which health care providers provide clear and appropriate health information.

According to the US Department of Health and Human Services [26], 90% of adults in the United States experience difficulty understanding health information that currently is available through the media, health care facilities, and communities. This struggle to understand health information or health illiteracy occurs when information is not provided in a patient's preferred language or if the way information is presented is not well suited for the patient [27]. Health literacy in not solely based on an individual's ability to read; it is their ability to read, listen, and analyze health information and then apply that information in order to make complex health care decisions [28].

When health care providers use medical terminology that patients are not familiar with, it may be misunderstood and can lead to difficulty following health care plans and treatment. Although limited health literacy can occur among any population, it is more prevalent among those who speak limited or no English, minority populations, medically underserved communities, and older adults [27]. Generally, people understand written information at a reading level three years lower than their highest level of education. For example, an adult who has earned a high school degree likely reads at a seventh or eighth grade education level [28].

*Health Literacy in HIV/AIDS*. As of 2013, 35 million people in the world are living with HIV; approximately 24.7 million (70.5%) of those living with HIV are located in Sub-Saharan Africa [29]. Despite efforts to decrease transmission rates and increase education and prevention efforts, HIV continues to spread. With 2.1 million new infections each year, HIV continues to be a worldwide concern [29]. Furthermore, approximately half (19 million) of those living with HIV do not know their positive status [29, 30]. Although these statistics are concerning, the rate of transmission has seen a steady decline over the past few years [30]. Between 2011 and 2014, the rate of new HIV infections declined by 13% globally; in 10 countries HIV new infection rates dropped by 75% and in 27 countries the rate dropped by 50% [30]. These decreasing rates can be attributed to education and prevention efforts, increased access to health care, and increased supply of antiretroviral medications [30].

In Sub-Saharan Africa, 86% of those who know they are infected with HIV have access to and are taking ART medication; most of those (76%) have even reached viral suppression [30]. However, this is not the global trend. Most PLHIV (63%) are not able to access ART [30]. There are many reasons a person may not know their diagnosis, establish a professional relationship with a health care provider, undergo treatment with ART, or adhere to HIV medications and treatment. These include lack of access to health care facilities or providers, inability to pay for testing or transportation, belief that treatment is unavailable or ineffective, culture and language barriers, fear of discrimination or stigma towards HIV or testing site, and confidentiality concerns [31–34].

Almost all of the previously mentioned barriers may be impacted by an individual's level of health literacy. Low health literacy has been linked to less understanding about HIV and its treatment, lower CD4 cell counts, higher viral loads, and more frequent hospitalizations [3, 4, 35]. Health literacy has been so strongly linked to disease understanding, treatment adherence, and health outcomes that recommendations suggest that health literacy should be assessed routinely when working with patients, specifically those who are infected with HIV [21, 35]. Multiple studies have established that medication adherence when taking antiretroviral therapy (ART) is imperative in achieving optimal treatment outcomes; the risk of low medication adherence is possible development of viral resistance and decreased medication efficacy [36–38].

Kalichman and colleagues [39] reported that 18–25% of PLHIV had low health literacy skills, which contributed to a higher rate of missed medication doses than patients with higher health literacy. These missed doses then lead to poorer viral response to treatment and poorer immune functioning than their higher health literacy counterparts [39]. Even if patients with HIV report they understand their treatment plan during their office visit, low health literacy may still cause missed doses, difficulty understanding medication labels, and inability to understand prescription warning labels [40, 41].

Medication adherence and disease understanding increase when patients receive information regarding their treatment plan that is tailored to their specific need [42, 43]. When patients have answers to specific questions and that information is provided in a way that is easy for them to understand, they are more likely to comply with their medication regimen [42]. However tailors health information requires a larger investment of time and effort than general health information [42]. In an effort to save time, providers are more likely to provide the same health information to all patients with the same disease, regardless of their health literacy or primary language.

Because medication adherence is so important, practitioners must also understand the variety of psychosocial characteristics that play a role in a patient's ability and decision to take medications as prescribed. Factors that increase adherence include positive attitudes towards medication, fewer depressive symptoms, and greater self-efficacy for taking medications [38].

Maddigan and colleagues [44] define medication management capacity (MMC) as the “cognitive and functional ability to self-administer a medication regimen as it has been prescribed” (p. 333). In order to do this, one must be able to identify the correct medication, dose, and time of administration each time they are required to take medicine. This process can become extremely time consuming and confusing when applied to medication management [45, 46].

*Influence of Language and Culture in Patients' Management of HIV*. Health and health behaviors are influenced by culture [47]. Defining culture is complex; according to Fiske [48], culture is “a socially transmitted or socially constructed constellation consisting of such things as practices, competencies, ideas, schemas, symbols, values, norms, institutions, goals, constitutive rules, artifacts, and modifications of the physical environment.”

HIV/AIDS stigma has been shown to be a barrier between patients accessing prevention and treatment services [49, 50]. Attitudes towards PLHIV are largely negative among the general US population and usually include negative feelings related to homosexual stigma and personal responsibility [49, 51, 52]. A variety of factors including health literacy, culture, level of self-efficacy, and attitude towards health behaviors influence the level of health information an individual may acquire at any point during their health care or throughout their life [53]. As a result, determining how to appropriately tailor health information towards specific populations is complex and multifactorial.

This paper focuses on perspectives of health care providers (HCPs) and professional care team members (PCTMs) about the role of language and culture in health literacy for PLHIV. This study is part of a larger study aimed at gaining further understanding of individual factors that assist a person living with HIV to manage their treatment regimen, specifically to adhere to medications, reduce HIV transmission risk behaviors, and manage HIV. A second aim was to determine the perception of HCPs and PCTMs regarding the health literacy needs of PLHIV. Health care providers were HIV medication prescribers while professional care team members were non-HIV medication prescribing health care providers.

## 2. Methods

### 2.1. Study Design

The study employed a descriptive, exploratory qualitative design to explore patient-provider communication and its related factors from the perspective health care providers and professional team members. It was envisaged that a qualitative approach would increase understanding of individual factors that help HIV positive persons manage their HIV.

### 2.2. Study Settings

The study settings were Botswana, Puerto Rico, and the US. Botswana had one site in Gaborone while US had seven sites in Cleveland, Ohio; New York, New York; Newark, New Jersey; Wilmington, North Carolina; San Francisco, California; Boston, Massachusetts; and San Juan, Puerto Rico. Botswana represented a developing middle-income country in Southern Africa while the USA represented a developed high income country. The two countries have different health care systems, cultures, and languages.

## 3. Human Subjects Protocol

Ethical clearance was obtained from the University of California San Francisco institutional review board since the study was conducted as part of the UCSF International HIV Research Network. Additional review was conducted at each of the academic and clinical institutional review boards in each participating country and city. Individual participants provided written consent prior to participation in the study and were informed that they could refuse to participate or withdraw participation at any time.

### 3.1. Data Collection Tools

The sociodemographic data collection tool for PLHIV consisted of demographic and social characteristics of gender, biological sex, age, race/ethnicity, primary language, formal education, geographical residence, income, health insurance, employment status, duration of HIV seropositivity, AIDS diagnosis, and other medical diagnoses. The sociodemographic data collection tool for health provider consisted of the site codes, the demographic and social characteristics of age, nationality and residence, primary language, profession/cadre, gender, and HIV and AIDS management training.

Health literacy was addressed in separate focus groups: those with PLHIV and those with health care providers and professional care team members. In the PLHIV focus groups, 11 open-ended questions covered information on HIV and its treatment, the nature of patient-provider communication, and facilitators and impediments for accessing HIV-related information. For the analysis in this paper, we conducted focus groups with health care providers and professional care team members and included six open-ended questions related to HIV information for patients and challenges in providing HIV and treatment information to patients. The questions regarding challenges were organized into three topics: the patient's own personal factors, environmental factors, and health care system factors.

## 4. Population and Sampling

The populations for the study included professional care team members and health care providers managing the treatment regimen for people living with HIV. Health care providers included prescribers and nonprescribers of HIV medications.

A convenience and purposely drawn sample of 32 health care providers and 39 professional care team members was included in the focus groups from all study sites. PLHIV and health care providers were organized into 17 and 6 focus groups, respectively. Informed consent was obtained from the HCPs and PCTMs with discussion of the purpose of the study by the principal investigator or study staff. Health care providers/health care professionals included in the sample were required to be providers of direct care or support to PLHIV.

### 4.1. Recruitment of Study Participants

Participants were recruited from a total of eight sites: one site in Botswana and seven sites in the USA. Participants were recruited because of their involvement in direct care provision in HIV clinics and AIDS Service Organizations. Data collection occurred during the period of June to December 2013. In each site, the principal investigators or a study staff explained the purpose of the study and provided the consent form. Upon completion of the consent form, the focus groups were held. Remuneration was offered in the form of a small monetary token of appreciation.

### 4.2. Data Collection

Quantitative data were collected by the investigators from individual participants in a private space with the aid of a structured tool including demographic data. The participants responded to the tool in the presence of an investigator who clarified questions. Focus group discussions were used to generate qualitative data from the health care providers and professional care team members. The discussions were facilitated by the investigators with the use of an open-ended interview guide. The focus group discussions were conducted in private quite rooms and lasted 1.5 to 2 hours. In all sites, the principal investigator(s) observed the process and took notes throughout the interview session. The discussions were audiotaped and later were transcribed verbatim.

## 5. Data Analysis

Descriptive statistics were used to analyze the demographic data. Transcribed data from the focus group discussions were imported into ATLAS.ti qualitative analysis software (ATLAS.ti, 2011) for our approach to coding and analysis. The analyses were developed by working through each transcript, development of a codebook, and double-coding several transcripts to ensure intercoder reliability (90%). Subsequent to the development of the codebook, a single coder with expertise in qualitative methods completed all coding. Content analysis was conducted with review of all coding and themes that were identified in ATLAS.ti by the principal investigators from each site.

## 6. Sample

### 6.1. Characteristics of the Sample

#### 6.1.1. Health Care Providers (HCPs)

Health care providers were HIV treatment prescribers and they were 32 in number (35.3% female and 64.7% female valid entries) from 6 USA sites (56.2%), one Botswana site (25%), and one Puerto Rico site (18.8%). The sample of health care providers had a mean age of 45.4 years (SD = 10.2) with a range of 28 to 63 years. Primary languages spoken were primarily English (65.6%) and Spanish (18.8%). Highest educational qualifications were doctoral/medical/law degree (43.8%), master's degree (40.6%), college/BS or BA (12.5%), and others such as Doctor of Nursing Practice (DNP) and nurse practitioner (NP) (3.1%). Professional titles were advanced practice nurse (45.2%), physician (34.4%), registered nurse (9.7%), and others such as dentist and physician assistant (9.7%).

#### 6.1.2. Professional Care Team Members (PCTMs)

Professional care team members were non-HIV medication prescribing health care providers. The sample included an *n* of 39 (72.7% females and 27.3 males) from 5 USA sites (92.1%) and one Botswana site (7.9%). The sample had a mean age of 42.7 years (SD = 11.1) with a range of 25 to 72 years. Primary languages spoken were English (86.8%) and others such as Setswana and Portuguese (13.2%). Highest educational qualifications were two-year college programs (33.3%), college/BS or BA (33.3%), master's degree (30.8%), and doctoral/medical/law degree (2.6%). Professional titles were registered nurse (39.5%), case manager (10.5%), licensed social worker (7.9%), and a variety of others such as counselor, chaplain, and medical assistant (41%).

## 7. Results

This study examined health literacy and the perspectives of PCTMs and HCPs. Several themes that emerged from the data addressed aspects of culture, language, health seeking behaviors, and traditional healing/complementary and alternative healing.

### 7.1. Ineffective Communication Related to Language and Culture

Ineffective communication included issues of spoken language, use of technical language, and nonverbal or body language. Spoken language was problematic to both patients and providers. Some patients did not understand the language of the HCP while some HCPs did not understand the language of the patient. The issue of ineffective communication arose in 5 out of 9 sites where focus groups were conducted. For instance, in Botswana, most health care providers/physicians were foreigners who could not speak the local language while many of the patients could not communicate in English. In San Francisco, California, 7% of the patients spoke Spanish while others spoke French, while the main stream language was English. Ineffective communication at times manifested through the patient's nonverbal or body language upon arrival for inclusion in the study.

One health care provider reported on language barriers and interpreters as follows:

*Language barrier is a challenge, yes it is another one, the other one would be language, sometimes they don't explain properly even when the interpreter is there, you don't know what the interpreter is saying the right thing, you may say something in a minute and the interpreter takes five minutes and you wonder what the interpreter is saying (lots of laughter from both researchers and doctors) and you are thinking what is she going to say?*

The ineffective communication was addressed through the use of several strategies. The commonest strategy in different sites was the use of interpreters. The concept of interpreters varied across sites. High resourced countries such as the USA are required to use professional interpreters in many settings and may use language telephone lines and iPad devices. However, even in the US, not all sites could afford professional interpreters (if they were not required by statute to do so) and resorted to using family members though this is a known transgression of ethical standards. Even those who had interpreters did not have interpreters available in all languages. While the health care providers and professional care team members appreciated the assistance that was present in some health care systems for interpretation, they also noted the limitations of this service. One health care provider had this to say:

*None of the physicians or providers like using the language line, because it is so impersonal, but we do not have interpreters for fifty languages. I know that we're seeking a case manager that might be bilingual, but it's not easy to find. They just kind of work together with interpreters, case managers, and we just try to seek out what we can, but it's tough. It's not ideal. *

In Botswana, which is a low resourced country, staff used for interpretation were nontrained staff and family members. Interpreters were particularly used at the initiation of treatment where comprehensive assessment was conducted, as one PCTM noted the following:

*Yes, your interpretation is correct, you rely on the interpreter. Especially when you are initiating [Anti - retroviral therapy] because that's when you ask more questions, at the beginning.*

Physical disability also posed a communication barrier, as one health care provider noted:

*I have a couple of patients who have HIV and who are deaf, and so that presents a different [issue]. I think this goes back to the other thing about cognitive ability.*

Even where health providers were able to communicate with PLHIV in the same language, it was difficult for some patients to grasp the concepts that they were expected to understand. One health care provider who had been providing care to a patient for years noted the challenges of using analogies that make sense to patients with low literacy, given the microscopic size of the virus.

*I've always found that talking about HIV to people with low health literacy, people who basically don't understand much about how the body functions is very difficult because it is completely invisible, the virus is invisible, the T-cells are invisible, no one's ever heard of a T-cell before and I don't think I've ever found a way that really feels effective to talk to it about it to people who are of that level. I had one experience, I had a patient who I was taking care of for years, and I sort of thought she got the general picture and she went to see H and he just told me “she doesn't have a clue.” And, kind of discouraging for me because I speak Spanish and I thought I was explaining to her using the language that she could understand, but I wasn't. And H somehow built a house out of bricks and used some analogy of bricks and a house that may have worked better than any analogy I ever tried to explain.*

Although the nurse was fluent in Spanish, he did not grasp the importance of developing a creative way to enable the patient to understand the medical concepts under discussion. At the same time some providers lacked enthusiasm for information and were not willing to seek more evidence. Instead, they were just content with telling patients that they were told so and did not know how to use learning theory to support their HIV education, as one HIV provider stated:

* So when you teach them about this they will ask you, how are the risks, we do not know, we are just told, we are just given information, so we just tell them even us we don't know.*

During provider patient interactions, some providers used medical jargon that was not understood by PLWHIV. From some providers' perspectives, they noted that there was a spectrum of health literacy levels, with some patients being illiterate, while others had limited literacy and some although educated lacked literacy related to medical jargon. It was also apparent that people with low health literacy found it more difficult to understand the medical jargon than those with high literacy levels. Health care providers also had difficulties with expressing themselves in a simpler way and in the patients' primary languages. This was, however, limited since health literacy was not directly measured in this study, language.

One health care provider reported the following:

*I asked him, do you know what a T-cell is or CD4 cell is? He said no, so we are starting all over, and he's still not taking his meds. *

Another RN noted the need for nurses to clarify instructions to clients at a later date since during consultations patients are inundated with lots of information in a short span of time, leading to confusion as reflected below.

*The provider had a tendency – and he knew it too, - but to use a lot of medical jargon, you know, to fly through a lot of information very quickly, and so having the nurse be in the room, who then can, either after the visit or next week, or you know, whatever, go through, like, “Hey, did you really understand what so- what your provider was telling you?” Or they say, “I heard I'm getting 60 Percocet next week,” and I'll say, “Well, no, actually I heard that you're not getting those for another six weeks.”*

### 7.2. Health Seeking Behaviors

Cultural orientation to health seeking was shaped by the context in which the PLHIV had been raised. Some patients had been raised under episodic care coupled with the use of home remedies or traditional/complementary healing, and approaches to western medicine were unfamiliar to them. One provider noted the following:

*Some of these folks don't have that, have experience in that,… have experience in some Latin American countries of just episodic care. So to think that you have an illness that needs ongoing, lifelong care is a new concept altogether.*

Therefore, expecting patients to conform to this process and adhere to treatment was a challenge as yet another provider noted the following:

*… A lot of the Spanish speaking people who don't have a concept of what continuity of care is. And it's more episodic [care].*

### 7.3. Cultural Facilitators

Cultural barriers and facilitators influenced the health literacy experience of PLHIV in several ways. Cultural beliefs informed health seeking behaviors and the way PLHIV responded to the diagnosis and treatment of HIV. Evolution of cultural beliefs was evident among some PLHIV as was illustrated by patients who made an effort to repattern their beliefs. Health care providers also described efforts to address the integration of culture for their patients.

Culture evolves over time and this was illustrated by some of the patients who had been on treatment for a while. Some health care providers also noticed the changing cultural beliefs of PLHIV. Cultural transition was noted by both health care providers and PLHIV. One health care provider noted the following:

*To me I would say culture is not much of a problem, because people now understand HIV even more, because in the past it used to be associated with widowhood and the likes, but I would say nowadays culture is not much of a problem, I think maybe it's because of the pill fatigue, but culture is not a problem nowadays, even though some of them would start with traditional doctors before coming here, but people do understand.*

Understanding the importance of culture in health literacy and transcending language was illustrated through the quote of this PCTM:

*Sometimes I would try to draw things, or, especially with patients where English wasn't their first language and I, and we didn't have access to let's say a Creole interpreter, or the person I work with wasn't available, especially if I was doing like, just like medication teaching, I would actually, like, get the pills, and so then they could, you know what I mean, or their medications, and we'd go over them kind of in a hands-on approach, so they knew, like, the colors and what they were working with and things like that.*

In the Botswana setting, one provider noted the following.

Health care providers in cognizance of the cultural beliefs of the PLHIV employed caring strategies that reflected cultural competence. Aware of the pivotal role of cultural beliefs in the health literacy of PLHIV, health care providers used strategies that reflected cultural competence on their part. They selected approaches that were more successful in dealing with the patients in their homes. This health care worker noted the importance of contextualizing care to the culture of the patient.

*Each culture has different strengths in faith or mores or whatever. If you don't use it, you can't help the person get better. Or you can't help the person to make a plan to get better. If you think that I'm medical and I'm all that, and I have it covered, you are a fool. They have to know that every nuance of their being has to come into play in order for them to want to get better, or I'm not being punished for something, what did I do in my life to deserve this, and so you can refocus them and get them around but you have got to use what is important to them in order to get them there. *

Another health care worker noted the importance of going into the client's environment to establish rapport.

*It's been my experience, and I've had quite a few clients, I would go to their homes and they would say my CD4, my levels are this, and I only take this herbal medicine, I never take my pills. So, based on their religious beliefs and what they really believe, you'll get the real story when you're sometimes outside the clinical setting. And sometimes in the clinical setting, if they've felt warmth to the providers, the nurses, or the physicians, they will even share with them their religious preferences or even ask them to pray for them. *

Health care systems also responded positively to issues of cultural diversity by arranging knowledge development seminars on culture of the patients for their staff. One health worker reported what the management was planning to do.

*At our next network meeting, El Puente, a Hispanic agency, will be talking about culture, and population, and the services they provide for the Hispanic population in hopes that it would lead to an in-service for providers to be more interested and go to regarding the Hispanic population and HIV [in order to provide optimal care].*

Cultural competence was therefore observed at a health provider and management level.

### 7.4. Traditional Healing/Complementary and Alternative Modalities (CAM)

In addition, these patients frequently used traditional healing practices or complementary and alternative care therapies such as herbal medicines and spiritual healing. Concerns were voiced by health care providers stated that

*Spanish speaking people use a lot of herbal remedies or other remedies that [um] have actually worked for them in the past so why would they try some of these pills that make them very ill sometimes? *

Yet another health care provider in the US reported the following:

*I am just thinking of a couple of patients that are from Haiti or outside the U.S., and previous knowledge based on whether it's popular myth or family discussions is really hard to dissuade. I have a couple of patients who adamantly do not believe that they have HIV, or that they, you know, can treat it with medicines that they get at home, and so, I don't, I don't have an answer for that. I don't know how to fix that or change that or talk to it.*

Indeed, a few patients consulted a traditional healer before being diagnosed with HIV in a clinic. Some patients admitted consulting traditional healers to no avail and after approaching the western health care system where a diagnosis of HIV positive was made, followed by initiation of ARVs, one patient had this to say:

*I was losing weight and had persistent headaches. I used to smoke umm…what is it? …I used traditional medicine and it was not helping. Then I said to myself, we are told about the incurable disease from the radio and we are urged to go to the hospital and get tested, I went to the hospital in 2009 and got tested and checked my CD4 count and my CD4 count was 400.*

Another health care provider in the US noted the following regarding complementary and alternative methods used by PLHIV as an adjunct to ARV therapy:

*What I do is you still have to give them the information that you know is valid. Whether they receive it or not, or if they want to put their belief as primary for what's healing them, or what they feel as though is going to be their punishment, or whatever their beliefs and customs. Sometimes I just stick to what I know is valid, and you listen. Sometimes it's the opportunity to just allow them to talk about what their beliefs and practices are, and then just to come alongside them. You can't really go against it. *

## 8. Discussion

The results of this study suggest the critical importance of cultural and linguistic barriers and facilitators to health care and the impact of health seeking behaviors and traditional and complementary/alternative healing. Several strategies were used by health care providers to render care in the face of these barriers with some strategies yielding positive outcomes for patients. When health care providers are not able to communicate in the client's language, quality of care and patient outcomes may be impacted.

In this study, participants described the challenges and some facilitators related to issues of culture, language, and literacy. Ineffective communication was noted to be a barrier—particularly related to spoken language, technical and medical jargon, and body language. Across cultures and settings in this study, language barriers existed. Not surprisingly, the use of interpreters served as a bridge to addressing health literacy according to the focus group participants. It is interesting to note that in Botswana, where the use of medical interpreters may be more difficult and not mandated as in settings in the US, that nontrained staff and family members served as interpreters.

The need for creative approaches to overcoming barriers related to health literacy was described by the health care providers and professional care team members. Teaching tools and web-based resources are important approaches that have merit for addressing low literacy. Use of medical jargon was viewed as a major barrier to overcoming challenges to health literacy by many participants in the study.

The theme related to health seeking behaviors was associated with linguistic and cultural barriers by participants. They noted that cultural orientation to health seeking varies across cultures. For example, several cultures were identified as focusing on episodic rather than primary care. Also HIV emerging as a health challenge may not have occurred in the primary care setting but rather as a diagnosis due to health issues late in diagnosis. In some settings, health care providers and professional care team members discussed the importance of integrating traditional healing and complementary therapies with western medicine approaches.

Cultural facilitators were linked to health literacy according to many study participants in the focus groups. Many health care providers and professional care team members identified integration of culture within the diagnosis of HIV disease and in the plan to address literacy issues. They also noted that culture evolves over time and that changing cultural beliefs were a subtheme whereby HIV was integrated into the culture of living with HIV. In the Botswana setting, the participants noted that being cognizant of cultural beliefs in working together with those living with HIV disease was pivotal to optimal HIV care. The issues of contextualizing and individualizing care as well as understanding the environment and religious beliefs of patients with HIV were identified as key to success in caring for those living with HIV. The role of health care settings in responding to cultural diversity and developing educational sessions that are inclusive of culture and literacy issues are also important.

Traditional health and complementary/alternative modalities were an important theme identified by participants—particularly herbal medicines/therapies and spiritual healing. The importance of the role of the traditional healer within cultures and having a shared cultural “language” related to healing is critical for health care providers and professional care team members to understand. The integration of traditional healing and western medicine emerged as an important focus as noted by several participants across all settings. The importance of providing valid, high quality education within a framework that is culturally sensitive and at the appropriate level of literacy for those living with HIV is a critical area for further investigation.

Traditional beliefs about HIV and AIDS and poor health literacy have been reported as barriers to entry to HIV care and adhere to ART in Southern Africa [54]. In this study, traditional beliefs and low literacy were also identified by health care providers as barriers to nonadherence to ART. An ongoing and individualized assessment of culture, language, and level of health literacy is required in all health settings that provide HIV care. Patient-specific interventions by all health care providers and professional care team members are critical to address the challenges and to enhance facilitators to optimal HIV care across the trajectory of the disease.
